# Laser Beam Drilling of Inconel 718 and Its Effect on Mechanical Properties Determined by Static Uniaxial Tensile Testing at Room and Elevated Temperatures

**DOI:** 10.3390/ma14113052

**Published:** 2021-06-03

**Authors:** Jana Petrů, Marek Pagáč, Martin Grepl

**Affiliations:** 1 Department of Machining, Assembly and Engineering Metrology, Faculty of Mechanical Engineering, VSB—Technical University of Ostrava, 708 00 Ostrava, Czech Republic; jana.petru@vsb.cz; 2 Honeywell International s.r.o., 148 00 Prague, Czech Republic; grepl.martin@gmail.com

**Keywords:** laser drilling, recast layer, microcracks, Inconel 718, mechanical properties

## Abstract

Particularly in the aerospace industry and its applications, recast layers and microcracks in base materials are considered to be undesirable side effects of the laser beam machining process, and can have a significant influence on the resulting material behavior and its properties. The paper deals with the evaluation of the affected areas of the Inconel 718 nickel-base superalloy after its drilling by a laser beam. In addition, measurements and analyses of the mechanical properties were performed to investigate how these material properties were affected. It is supposed that the mechanical properties of the base material will be negatively affected by this accompanying machining process phenomenon. As a verification method of the final mechanical properties of the material, static uniaxial tension tests were performed on experimental flat shape samples made of the same material (Inconel 718) and three different thicknesses (0.5/1.0/1.6 mm) which best represented the practical needs of aerospace sheet metal applications. There was one hole that was drilled with an angle of under 70° in the middle of the sample length. Additionally, there were several sets of samples for each material thickness that were drilled by both conventional and nonconventional methods to emphasize the effect of the recast layer on the base material. In total, 192 samples were evaluated within the experiment. Moreover, different tensile testing temperatures (room as 23 °C and elevated as 550 °C) were determined for all the circumstances of the individual experiments to simulate real operation load material behavior. As a result, the dependencies between the amount of the recast layer and the length of the microcracks observed after the material was machined by laser beam, and the decrease in the mechanical properties of the base material, were determined.

## 1. Introduction

Nickel-based alloys are the most used alloys in the aerospace industry and its components as they can offer a higher chemical resistance, mechanical strength, and thermal conductivity compared to steels, for example [1,2]. Moreover, a great opportunity has arisen to produce parts for the aerospace sector with advanced performances made of nickel-based superalloys [3]. However, the manufacturing of parts from nickel-based superalloys currently represents a challenging task for industrial sites [4].

Due to their excellent properties, these superalloys are very difficult to machine conventionally and the final surface integrity of a machined component can be affected [2,5,6].

In particular, the conventional drilling process of effusion cooling holes in a combustion chamber made of Ni-based alloys has reached its technological and economical limits. Nonconventional machining technology, such as laser beam drilling, could therefore be a cost-effective alternative for these materials and will be more important in the future [7].

When speaking of conventional machining processes, the research performed by Pusavec et al. [8,9] using the Inconel 718 alloys revealed that the application of MQL (Minimum Quantity Lubrication) can reduce the cutting forces, tool wear, and chip breakability and can increase productivity. In fact, this observation is not a sufficient argument for the possibility of comparing the drilling efficiency with a laser.

When taking into consideration the fact that thousands of effusion cooling holes are drilled on to the combustion chamber with a very thin wall, conventional drilling by a drilling tool would be inefficient, uneconomical, very time-consuming, and maybe even impossible.

The Electrical Discharge Machining (EDM) process has also been used for the machining of features onto aerospace components [10,11,12,13,14]. The new strategy in EDM milling allows the machining of complex geometries by using a standard cylindrical shape rotary electrode, which is used for the machining of complex shaped diffusors in Inconel 718 turbine blades. This manufacturing strategy was researched by Kliuev et al. [15]. The proposed strategy offers a 15% higher productivity in comparison to the layer-by-layer machining strategy. Another manuscript deals with the use of modern EDM drilling machines for drilling cooling holes and diffusers in turbine blades. Kliuev et al. have published studies that have shown where the material removal rate reached 77 mm^3^/min, the relative tool wear was reduced to 20%, the average recast layer thickness was reduced to 8 μm, and the roughness Sa of the internal surface was less than 1 µm. The EDM process is available for combination high-speed cutting with relatively low recast layer thickness and with very good surface quality [16]. In the future, aero engines will typically have an excess of 150,000 cooling holes [17]. This is very important for the research and development of the optimalization technology process. This manuscript is interesting as it contains a study of high-speed hole drilling with a diameter of 0.8 mm [17]. It is important that EDM technology has very good precision. Zou has presented research about the precision of EDM of a micron-scale diameter hole array using in-process wire electro-discharge grinding high-aspect-ratio microelectrodes. To improve machining accuracy, an in-process touch-measurement compensation strategy was applied to reduce the cumulative compensation error of the micro-EDM process [18]. However, due to reasons that also apply to the conventional drilling method, the EDM method is not comparable with laser beam machining due to the cycle time of the drilling process itself.

Laser beam drilling is currently widely used for various aerospace applications where high dimensional accuracy and hole quality are required [19,20,21,22,23]. This process is especially suitable for processing difficult-to-machine alloys by conventional machining processes [24]. In addition, a large number of holes can be produced by laser drilling in a non-contact manner (as opposed to conventional drilling methods). However, the process also has certain defects associated with the resulting drilled hole geometry [25,26] and material microstructure [17,27,28,29,30,31,32]. These are mainly heat affected areas, recast layers and microcracks in the base material, which are considered to be undesirable effects. In the real production process, it is recommended to minimize them. The residual stress and finite element method and an experimental analysis of residual stress and elevated temperature are also important [33].

Previous studies have already shown that for the laser drilling process, parameters such as pulse energy, pulse width, and pulse frequency are the most influential—all of these increase the recast layer thickness and lead to the formation of microcracks, as investigated by Morar et al [34].

In terms of the future, there are many opportunities that could focus on the field of Additive Manufacturing (AM) adoption as an advanced manufacturing technology for this kind of aerospace product. However, first there are many challenges in the field of AM which need to be investigated, such as the distortion, fatigue, defects, and residual stress of the manufactured parts [35].

For the Inconel 718 material, the influences of input laser energy density on densification behavior, phases composition, microstructures, microhardness, and wear performance of the AM as-built samples were explored by Liu et al [36].

This investigation has been performed with the purpose to describe the harmful effects, if there are any, of the laser beam drilling method when used in the processing of aerospace applications. It is known that during the process, the recast layer of the material is created and it adheres to the machined surface. In addition, the recast layer is known as a crack-into-the-base-metal initiator and so it was expected that the mechanical properties would be adversely affected as a result of this phenomenon. This was confirmed. This result is compared to conventional drilling by a drilling tool, where the recast layer is not expected to be observed and the mechanical properties of the base material should not be affected so significantly.

In the first section of this article, the specifics of laser drilled holes and the effects on the base materials are mentioned. The second section describes all the properties of the experimental samples, their processing, and the laser machine used in the experiment. The evaluation methodology, the achieved results, the equipment and the software used are given in section three of this article. The final discussion and all the conclusions obtained can be found at the end of the article.

### 1.1. Hole Shape Deformations

A typical shape deformation of a drilled hole by a laser is a taper [37]. The taper of the hole can be eliminated by optimal values of laser drilling parameters, but this will always depend on the material and the thickness to be drilled [38,39].

The taper of the drilled holes is caused by the removal of molten and vaporized material away from the hole. The percussion drilling strategy, used in this experiment, (several consecutive pulses, in a short time, that gradually create a drilled hole) is a complex process with a large number of variables. However, in general, a shorter pulse width means a more significant taper. On the other hand, the degree of the taper decreases with the increasing thickness of the drilled material.

Manufacturing processes, including laser drilling, used to drill effusion cooling holes on gas turbine engines currently, can result in some geometric deviations (e.g., conical angles, filleted edges, diameter deviations, etc.). This can reduce the cooling effectiveness, heat transfer performance, and aerodynamic characteristics of the final products [40].

The potential geometric deviations by laser drilling techniques were gathered by Bunker [41] and the report [42] was from PRIMA North America, Inc. It was pointed out that the statistical data of effusion cooling holes are subjected to a Gaussian distribution, even if the most advanced laser drilling techniques are used.

Outside the area in which the hole shape deformations are located, the efficiency of the engine is also dependent on other factors.

### 1.2. Recast Layer Formation

The formation of the recast layer (thickness in the order of μm) is one of the accompanying undesirable effects, which is influenced by the resulting state of the machined material. Due to the high surface energy density of the laser beam absorbed by the material and the fact that its energy is converted into heat, the surface melts and solidifies very quickly when the laser radiation ceases to act on the area of the material being machined [43]. A recast layer then forms on the surface when heat dissipates [44]. The newly formed microstructure of the recast layer has an amorphous (glassy) character. It is characterized by high hardness and brittleness and is therefore prone to cracking. The cracks spread along the least resistant path—along the grain boundaries. Cracks can then have a significant negative impact on the fatigue strength of aircraft engine components while reducing their durability and safety [45].

After the interaction with the laser beam, the material is affected in three layers. There is an oxide layer on the surface, which can be partially eliminated by the process gas (inert gases) used. This is followed by a recast layer of the base material which has solidified again on the surface to be treated and contains oxidic inclusions. Between the recast layer and the unaffected base material, there is a heat affected zone (HAZ) of the base material. Previously, Sezer et al. [46,47] experimentally observed a larger HAZ on the leading edge side and a larger recast layer on the trailing edge side of the hole.

The thickness of the recast layer can be reduced by the appropriate selection of process parameters. However, it is considered that a tiny layer will almost always occur on the machined material and its formation cannot be completely prevented due to the inevitable influence of the material with a huge amount of surface energy density. Nevertheless, these small volumes are unimportant to the technical practice because they do not initiate microcracks into the base material.

In practice, it is highly recommended, if possible, to remove the recast layer from the surface of the machined material by one of the machining methods that does not generate a recast layer, or any other process such as heat treatment (applicable in some specific cases only).

### 1.3. Microcracks in the Base Material

The formation of these cracks is initiated by the recast layer; therefore, it is important to analyze their length. These cracks can have a significant negative effect on the fatigue strength of the aircraft engine components, while reducing their life and safety [22].

**Figure 1:**
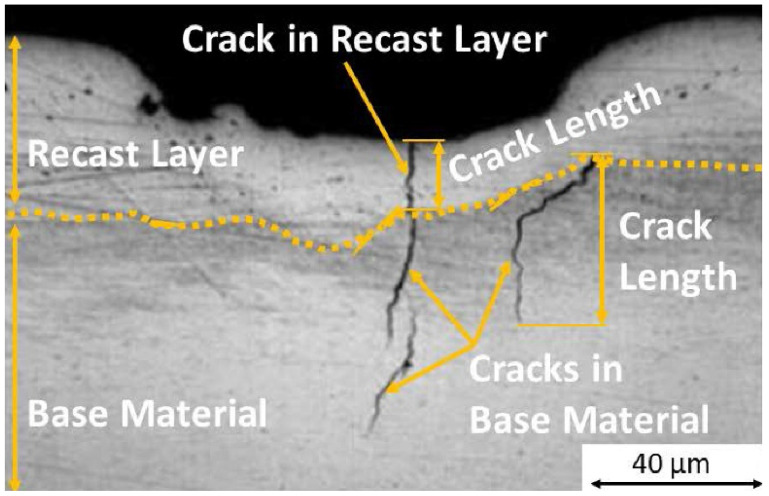
The crack formed in the recast layer and its propagation into the base material.

In Figure 1, the formation of a crack in the recast layer of the material and its subsequent spread in the base material can be observed.

## 2. Experimental Samples

### 2.1. Material and Thickness

High burning temperatures inside jet engines have made material selection complicated. That is why such materials require high strength and temperature resistance [16]. The test samples were made of high strength Inconel 718 alloy, which belongs to the group of nickel-base superalloys (Table 1). As high temperature resistant materials, these are suitable for operation under extremely demanding conditions. Nickel-based superalloys are generally used in industries such as aerospace, nuclear, etc. [48,49], and in applications such as land gas turbines and aircraft engine turbines. The Inconel 718 alloy is characterized by an excellent temperature resistance in the range of −253 °C to +705 °C, as well as an excellent oxidation resistance up to 980 °C.

Three different material thicknesses were chosen to cover the common range of thicknesses from which the real aerospace applications are manufactured: 0.5 mm; 1.0 mm; 1.6 mm.

**Table 1:** Nominal chemical composition of Inconel 718 [50].

Element	Fe	Cr	C	Ti	Mn	Si	Ni	S	P	Mo	Nb	Al
Weight %	17.62	18.84	0.024	0.95	0.02	0.06	53.64	0.002	0.03	3.08	5.23	0.53

### 2.2. Design, Amount, and Preparation

**Figure 2:**
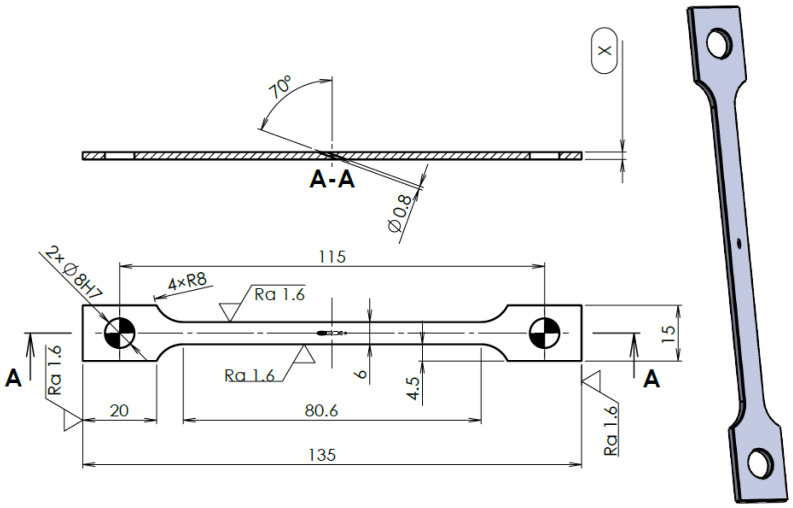
Design of the sample; all the dimensions in millimeters (X—sample thickness).

Flat shape samples were determined as the more suitable design to be used for the experiment. Except for their thickness, all the samples were manufactured with the same dimensions and under the same manufacturing conditions (Figure 2).

In total, 192 experimental samples were manufactured and evaluated (Table 2). There were four Sample Groups (1 through 4) including all three material thicknesses (A through C).

From a statistical evaluation point of view, and with regard to costs and time needed for manufacturing and the subsequent evaluation of all the experimental samples, 8 samples were established as the number of samples for each subgroup. They were expected to have the same condition.

At the end, there were two main groups of samples subjected to tensile testing. Each one of them contained all the material thicknesses and all the drilling methods previously applied, as well as undrilled samples (group 4) to reveal the basic mechanical properties of the material. The first half of the samples were subjected to tensile testing at room temperature (23 °C). The second half were subjected to tensile testing at an elevated temperature (550 °C).

Prior to the drilling of the samples, all were cleaned by immersion degreasing, and heat treated by solution annealing and precipitation hardening. Heat treating cycles are not specified as they are considered as intellectual property information. The reason was to have the material of the samples in the same condition as the material of real jet engine applications.

### 2.3. Drilling of Experimental Samples

The sample groups 1, 2, and 3 were drilled. The same rule was applied—one hole under a 70° incidence angle (a common angle of effusion holes) with a diameter of 0.8 mm was drilled into the middle of the sample length, either by a laser beam (groups 1 and 2), or by a drilling tool (group 3).

Effusion cooling holes (<1 mm) drilled by laser beam are highly valued for improving the performance of aviation engines [48]. It is envisaged that future generations of aero engines will typically have an excess of 150,000 cooling holes, which will result in enormous pressure on the technology used to meet targets in relation to productivity and hole quality [51]. Previously, Marimuthu et al. [52] investigated the effects of pulse duration, energy, frequency, and laser assisted gas composition on the characteristics of laser drilled holes. Kononenko et al. [53] also investigated the influence of laser pulses on the percussion drilling process, during which a dramatic rise of the recast layer thickness inside the drilled hole was observed.

The laser cycle is mainly determined by pulse width. The longer the pulse width, the longer the cycle.

To support the exothermic reaction, which helped to intentionally create more of the recast layer on the base material, oxygen was used as an assist gas to drill all the sample groups by a laser long cycle (1A, 1B, 1C).

As today’s fiber optic lasers offer the possibility to drill fast even at high qualities [54,55], the laser machine used for drilling was equipped with a fiber optic laser source with a maximum peak pulse power of 20 kW.

**Figure 3:**
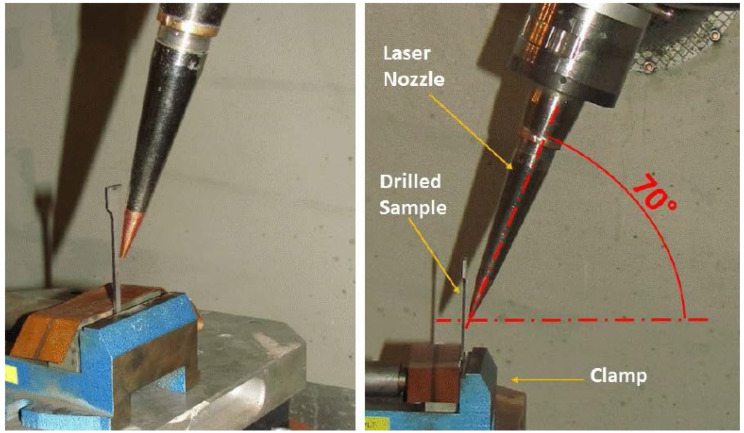
Clamped experimental sample to be drilled by laser beam—top left picture; laser nozzle adjustment.

Figure 3 shows the clamped experimental sample, as well as the nozzle position adjusted prior to the sample being drilled.

The samples were clamped by hand—however, repeatability of the drilling procedure was ensured by laser machine adjustment. Strictly speaking, the zero position of the laser nozzle was adjusted after each sample was clamped and so the position of the drilled hole and the drilling angle was always adjusted the same on each sample prior to its drilling. This is also the methodology used in the machining of the real application.

**Figure 4:**
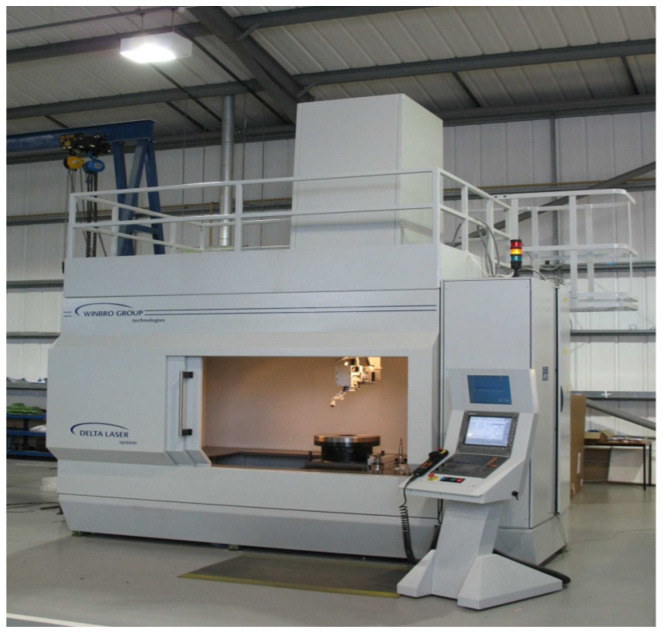
Left picture: Winbro Delta Tornado (Winbro Group Technologies, Coalville, UK), the laser machine used in the experiment [56].

The laser machine used for the drilling of the experimental samples can be seen in Figure 4. This machine is one of the most frequently used for the machining of aerospace applications by the contracting authority of this work, Honeywell Aerospace company.

**Figure 5:**
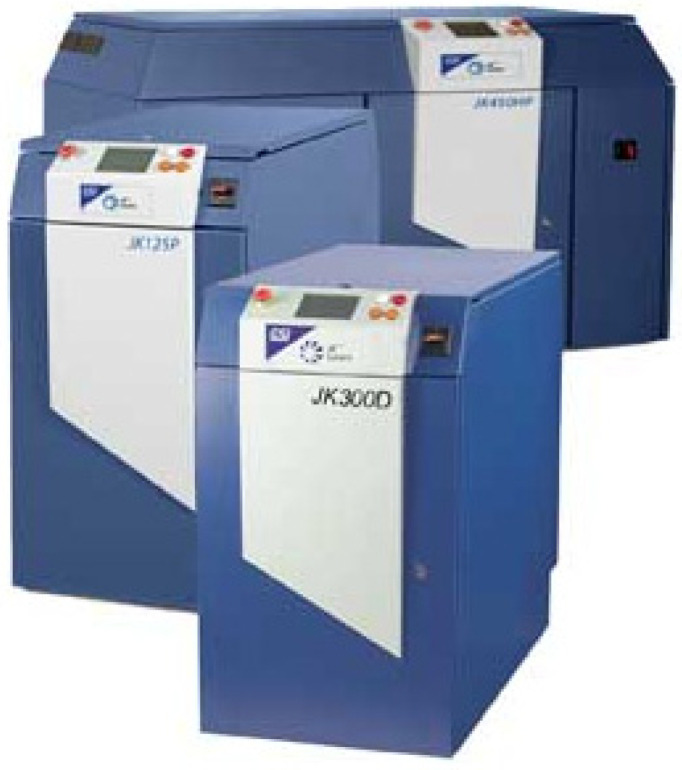
Right picture: Nd:YAG solid pulse source, type GSI JK (JK Lasers, Rugby, UK) used in the experiment [57].

The laser source, by which the laser machine was fitted, is shown in Figure 5. It is a solid-source type widely used for such processing. Currently, the solid-source lasers are being replaced by fiber optic laser sources, as these are more stable at the peak power and are easier to maintain.

In Table 3, all the process parameters (Assist Gas; Frequency; Pulse Width; Energy; Power; Number of Pulses) of a laser beam drilling process used on all applicable material thicknesses (0.5 mm; 1.0 mm; 1.6 mm) are described. Samples 1A, 1B, and 1C stand for the laser beam drilling with a long cycle, where more of the recast layer was expected to be formed. Oxygen assist gas was chosen to promote an exothermic reaction and the recast layer formation during the laser drilling. Samples 2A, 2B, and 2C stand for the laser beam drilling with a short cycle, where less of the recast layer was expected to be formed. Nitrogen assist gas was chosen to protect the melting area of the material during the laser drilling and to reduce the recast layer formation. The pulse width was also always higher for drilling with oxygen for the same reason. The number of pulses required to drill the hole increased with the increasing thickness of the sample material.

## 3. Results and Discussion

All the drilled samples were subjected to metallographic evaluation (Table 2).

### 3.1. Metallographic Evaluation of Drilled Samples

The two aspects of the metallographic evaluation were as follows:

1. Average thickness of the recast layer;
2. Length of cracks in the base material.

#### 3.1.1. Average Thickness of Recast Layer

The metallographic evaluation of the average thickness of the recast layer was always performed on each type of the drilled samples. Thus, it was performed for each drilling method used and material thickness. The recast layer thickness was measured in both longitudinal and transverse sections of the holes, shows in the Table 4. An evaluation of one section of the hole was performed in six equally spaced locations. It was expected that on the samples drilled by the laser beam, a recast layer would occur—whether for short or long cycle drilling. Surprisingly, a small amount of recast layer was also observed on the samples drilled conventionally, that is, by a drill. This, however, is negligible compared to the average thickness of the recast layer caused by the laser beam and it was not considered. In the aerospace industry, the upper limit of the average thickness of the recast layer is considered in the order of hundredths to tenths of a millimeter.

**Figure 6:**
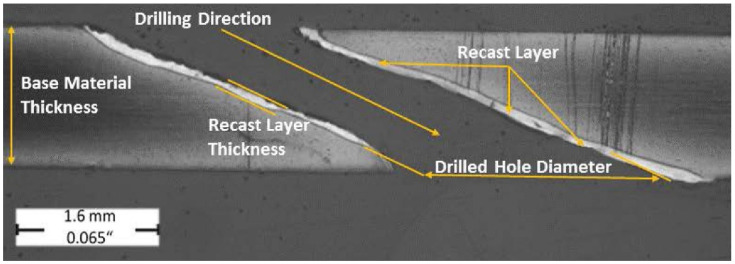
Longitudinal hole section (magnification 20x); sample group—1C (Table 2).

**Figure 7:**
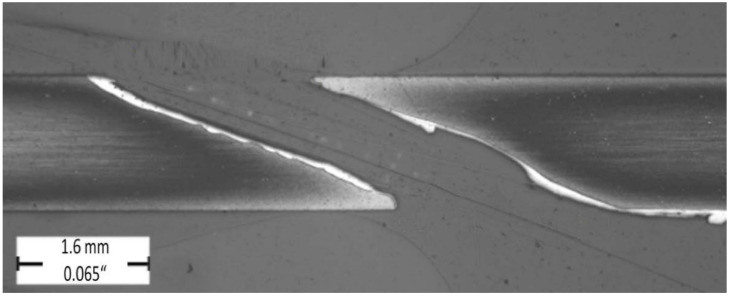
Longitudinal hole section (magnification 20x); sample group—2C (Table 2).

**Figure 8:**
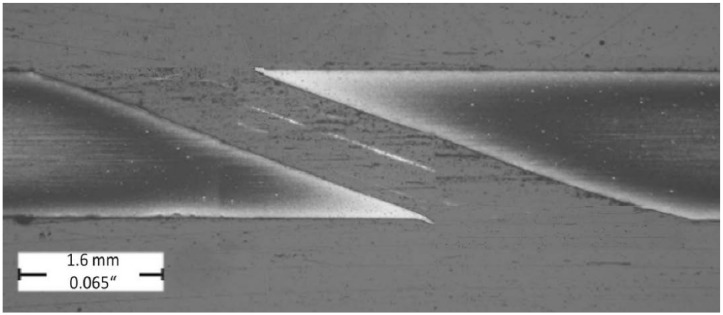
Longitudinal hole section (magnification 20x); sample group—3C (Table 2).

**Figure 9:**
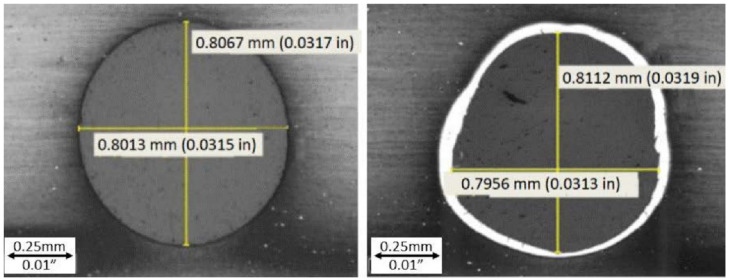
Transverse hole sections (magnification 50x); on the left: sample group—3C (Table 2); on the right: sample group—2C (Table 2).

The evaluated longitudinal hole sections are illustrated in Figure 6 and Figure 7 on which the formation of the recast layer along the hole drilled unconventionally by laser beam can be seen. The evaluated longitudinal hole section is illustrated in Figure 8 with a very slight and barely visible recast layer observed along the hole drilled conventionally by a drilling tool. The evaluated transverse holes sections are illustrated in Figure 9 to emphasize the difference between the hole drilled by the drilling tool and by the laser beam.

#### 3.1.2. Cracks into the Base Material

The cracks were also measured in both the longitudinal and transverse sections of the examined holes. The highest value, or the longest crack, was always recorded. In the aerospace industry, the upper limits of the length of the crack in the base material are considered in the order of hundredths of a millimeter.

As can be seen in Table 5, the highest values of the length of the cracks in the base material were always measured on the holes drilled by the laser beam using a long cycle (1A, 1B, 1C), when compared to the other drilling methods. The formation of the crack in the recast layer and the base material is shown in Figure 1.

### 3.2. Static Uniaxial Tension Tests

The first half of the samples (192 in total) were evaluated by a static uniaxial tension test at room temperature (23 °C), while the second half of the samples in total were evaluated by a static uniaxial tension test at an elevated temperature (550 °C). The reason for choosing 550 °C as the value of the elevated temperature was the fact that this is the temperature the base material is exposed to in real applications, such as jet engines, turbines, etc.

When evaluating identical samples by uniaxial tension testing at elevated temperature, the mechanical properties of the material are expected to decrease compared to the samples evaluated at room temperature. Thus, it was possible to compare the mechanical properties of the material, not only for the different drilling methods—thereby demonstrating the effect of the recast layer after laser beam drilling—but also the effect of room and elevated temperature on the tested material, with respect to its resulting mechanical properties.

#### 3.2.1. Tests at Room Temperature

**Figure 10:**
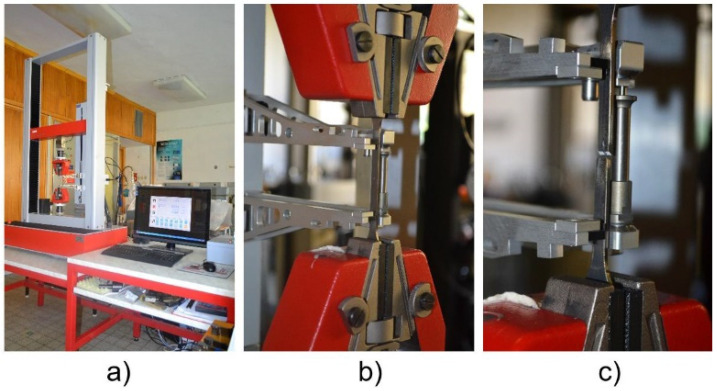
Static uniaxial tension test at room temperature: test cell (**a**); test sample prior (**b**); and after (**c**) the test.

The progress of room temperature tests as performed are shown in Figure 10a–c. The apparatus used to perform the tests is described below, as well as the main test parameters:

- Loading machine: M500-50CT (Testometric, Rochdale, UK)
- Software: WinTest Analysis (Testometric, Rochdale, UK)
- Test Temperature: 23 °C
- Loading Speed: 5.0 mm min^−1^.

Figure 10 shows the setup of the static uniaxial tension test at room temperature. The overall view of the test cell can be seen in Figure 10a. Figure 10b shows the loaded flat shape experimental sample with the extensometer set on the sample before starting the test, and Figure 10c shows the loaded flat shape experimental sample after the test was performed and the sample was broken, respectively.

In total, 96 experimental samples were evaluated by tests at room temperature. All these experimental samples were divided into four groups (see Table 2). Each sample group contained eight, theoretically identical, experimental samples evaluated under the same test conditions to ensure repeatability of the achieved results.

#### 3.2.2. Achieved Values of Mechanical Properties

In this section, the achieved values of the mechanical properties resulting from the tests at room temperature (E Modulus; Max. Load; Rp0,2; Rm; A50) are listed in Table 6, Table 7 and Table 8. The final values listed in the following tables were obtained from tests performed on eight experimental samples evaluated for each drilling method used (Laser Long Cycle; Laser Short Cycle; Drilling Tool; None). All the sample groups and total number of experimental samples are listed in Table 2.

**Figure 11:**
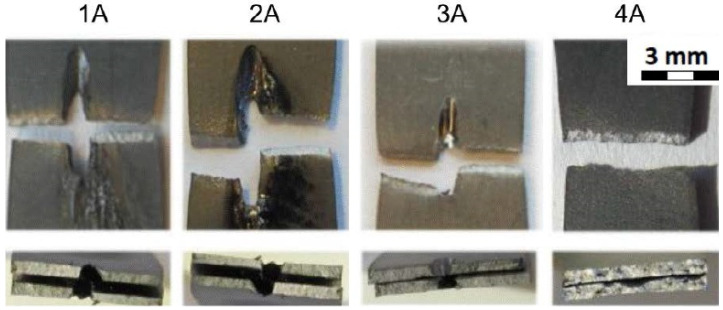
Fracture areas and fracture surfaces of individual sample groups—Material thickness 0.5 mm; Tests at room temperature.

**Figure 12:**
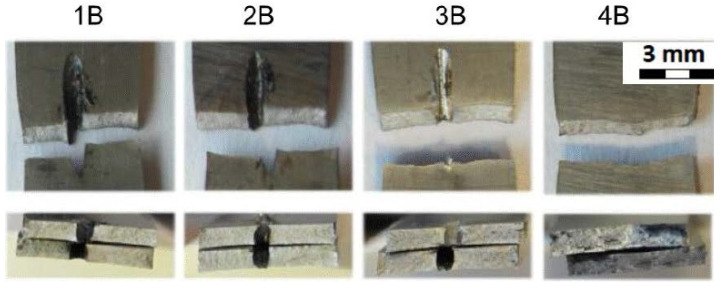
Fracture areas and fracture surfaces of individual sample groups—material thickness 1.0 mm; tests at room temperature.

**Figure 13:**
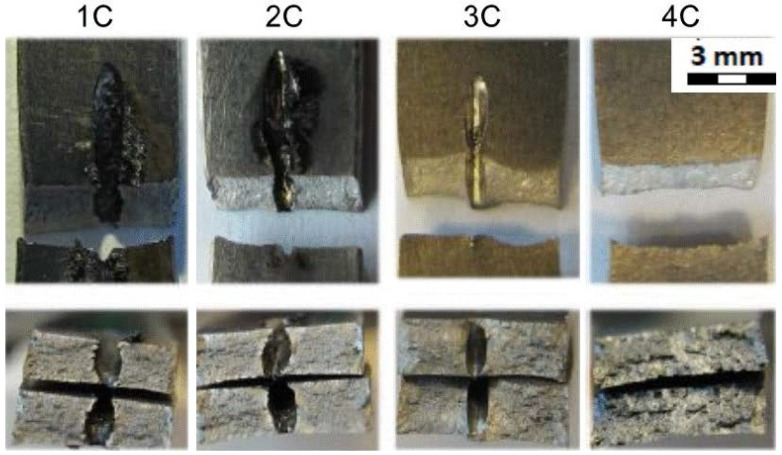
Fracture areas and fracture surfaces of individual sample groups—material thickness 1.6 mm; tests at room temperature.

In addition, the fracture areas and the fracture surfaces of the individual sample groups (1A–4A; 1B–4B; 1C–4C) are shown in Figure 11, Figure 12 and Figure 13.

The yield strength (Rp) is a numerical value of the non-proportional elongation in % (Rp0.2) and is usually indicated because (compared to the elastic limit), the yield strength can be determined by the stress–elongation curve.

The tensile strength (Rm) is a material characteristic value for the evaluation of strength behavior. It is the maximum mechanical tensile stress onto which a test specimen can be loaded. If the tensile strength is exceeded, the material fails (the test sample is broken).

Table 6 gathers all the values of the mechanical properties achieved by the static uniaxial tension tests at room temperature. All the sample groups with a material thickness of 0.5 mm are included (1A, 2A, 3A, 4A).

Figure 11 shows the fracture areas and the fracture surfaces of the individual sample groups. The top row of Figure 11 shows the top view of the fractured samples where the samples of 1A, 2A, 3A were drilled in the middle of their length. As can be seen, and as expected, the samples were broken in the area of the drilled hole. The samples from group 4A were not drilled and were broken approximately in the same area as the drilled experimental samples. The bottom row of Figure 11 shows the cross section of the fractured areas. In addition, the cross section through the drilled holes can be seen on the samples from the groups 1A, 2A, and 3A. The cross section of the sample 4A is for the samples that were not drilled at all.

Table 7 gathers all the values of mechanical properties achieved by the static uniaxial tension tests at room temperature. All the sample groups for the material thickness of 1.0 mm are included (1B, 2B, 3B, 4B).

Figure 12 shows the fracture areas and the fracture surfaces of individual sample groups. The top row of Figure 12 shows the top view of the fractured samples where the samples of 1B, 2B, 3B were drilled in the middle of their length. As can be seen, and as expected, the samples were broken in the area of the drilled hole. The samples from group 4B were not drilled and were broken approximately in the same area as the drilled experimental samples. The bottom row of Figure 12 shows the cross section of the fractured areas. In addition, the cross section through the drilled holes can be seen on the samples from the groups 1B, 2B, and 3B. The cross section of the sample 4B is for the samples that were not drilled at all.

Table 8 gathers all the values of the mechanical properties achieved by the static uniaxial tension tests at room temperature. All the sample groups for the material thickness of 1.6 mm are included (1C, 2C, 3C, 4C).

Figure 13 shows the fracture areas and the fracture surfaces of the individual sample groups. The top row of Figure 13 shows the top view of the fractured samples where the samples of 1C, 2C, 3C were drilled in the middle of their length. As can be seen, and as expected, the samples were broken in the area of the drilled hole. The samples from group 4C were not drilled and were broken approximately in the same area as the drilled experimental samples. The bottom row of Figure 13 shows the cross section of the fractured areas. In addition, the cross section through the drilled holes can be seen on samples from the groups 1C, 2C, and 3C. The cross section of the sample 4C is for the samples that were not drilled at all.

#### 3.2.3. Test at Elevated Temperature

**Figure 14:**
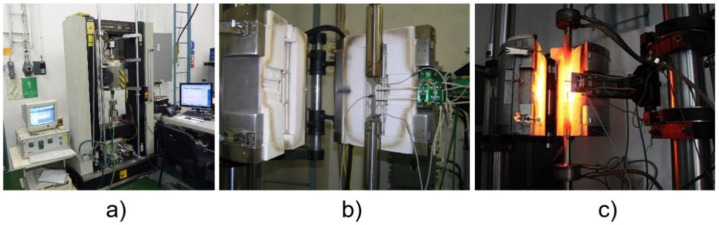
Static uniaxial tension test at the elevated temperature: test cell (**a**); test sample prior (**b**); and after (**c**) the test.

The progress of the elevated temperature tests, as performed, is shown in Figure 14a–c. The apparatus used to perform the tests is described below, as well as the main test parameters:

- Loading machine: INSTRON 55R1185-100 kN (Instron, Norwood, MA, USA)
- Control system: SYSTEM ID 5500 K5178 (Instron, Norwood, MA, USA)
- Extensometer: Epsilon High Temperature extensometer, model 3548-025M-100-ST of a 25 mm gauge length (Epsilon, Irving, TX, USA; Jackson, Denver, CO, USA)
- Furnace: 3-Zone Split Tube Furnace, ATS Model 3210. Operating range up to 1100 °C (Andy Group, Zhengzhou, China)
- Software: Bluehill^®^2 Software (Instron, Norwood, MA, USA)
- Strain control: actuator LVDT
- Test Temperature: 550 °C
- Loading Speed: 5.0 mm.min^−1^

The split tube furnace temperature was controlled by its controller system and the sample temperature was measured by a separate measuring system. The variation in the indicated temperature was ±3 °C. The time of holding on the specified temperature prior to the test was empirically established as 15–20 min. The samples were fixed in grips by the pins.

Figure 14 shows the setup of the static uniaxial tension test at the elevated temperature. An overall view of the test cell can be seen in Figure 14a. Figure 14b shows the loaded flat shape experimental sample with the extensometer set on the sample before starting the test inside the furnace, and Figure 14c shows the loaded flat shape experimental sample after the test was performed and after the sample was broken, respectively, in the heated furnace.

In total, 96 experimental samples were evaluated by tests at the elevated temperature. All these experimental samples were divided into four groups (see Table 2). Each sample group contained eight, theoretically identical, experimental samples evaluated under the same test conditions to ensure repeatability of the achieved results.

Figure 14 shows the set-up of the static uniaxial tension test at room temperature. An overall view of the test cell can be seen in Figure 14a. Figure 14b shows the loaded flat shape experimental sample in a tube furnace with the extensometer set on the sample before starting the test, and Figure 14c shows the loaded flat shape experimental sample after the test was performed and the sample was broken after its heating to the desired temperature (550 °C), respectively.

#### 3.2.4. Achieved Values of Mechanical Properties

In this section, the achieved values of the mechanical properties that resulted from the tests at the elevated temperature (E Modulus; Max. Load; Rp0,2; Rm; A50) are listed in Table 9, Table 10 and Table 11. The final values listed in the following tables were obtained from tests performed on eight experimental samples evaluated for each drilling method used (Laser Long Cycle; Laser Short Cycle; Drilling Tool; None). All the sample groups and total number of experimental samples are listed in Table 2.

**Figure 15:**
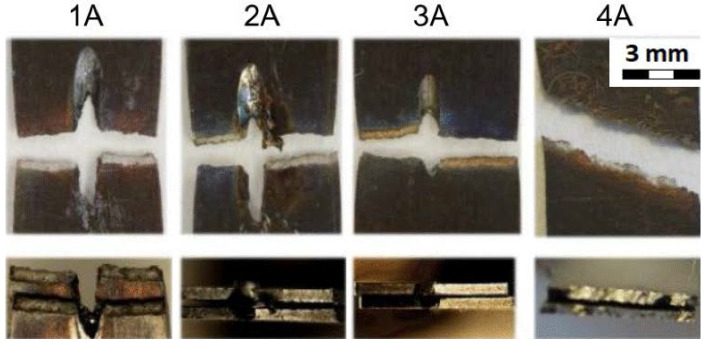
Fracture areas and fracture surfaces of individual sample groups—material thickness 0.5 mm; tests at elevated temperature.

**Figure 16:**
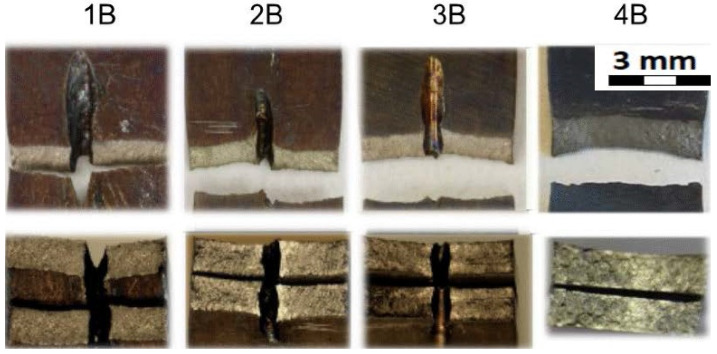
Fracture areas and fracture surfaces of individual sample groups—material thickness 1.0 mm; tests at elevated temperature.

**Figure 17:**
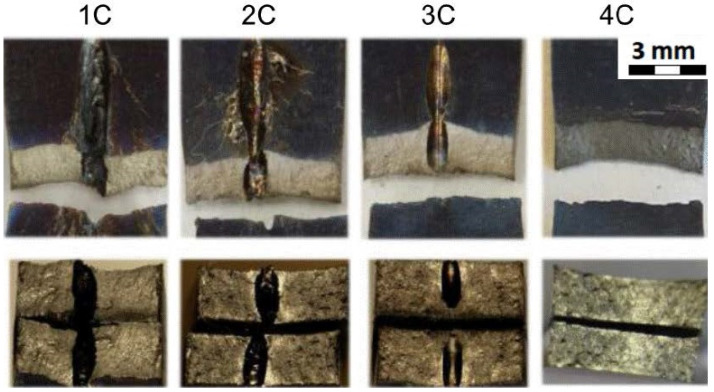
Fracture areas and fracture surfaces of individual sample groups—material thickness 1.6 mm; tests at elevated temperature.

Moreover, the fracture areas and the fracture surfaces of the individual sample groups (1A–4A; 1B–4B; 1C–4C) are shown in Figure 15, Figure 16 and Figure 17.

Table 9 gathers all the values of the mechanical properties achieved by the static uniaxial tension tests at the elevated temperature. All the sample groups with a material thickness of 0.5 mm are included (1A, 2A, 3A, 4A).

Figure 15 shows the fracture areas and the fracture surfaces of the individual sample groups. The top row of Figure 15 shows the top view of the fractured samples where the samples of 1A, 2A, 3A were drilled in the middle of their length. As can be seen, and as expected, the samples were broken in the area of the drilled hole. The samples from group 4A were not drilled and were broken approximately in the same area as the drilled experimental samples. The bottom row of Figure 15 shows the cross section of the fractured areas. Moreover, the cross section through the drilled holes can be seen on the samples from the groups 1A, 2A, and 3A. The cross section of the sample 4A is for the samples that were not drilled at all. In addition, the typical staining of the samples due to their testing at elevated temperatures can be seen here when compared to the samples evaluated at room temperature.

Table 10 gathers all the values of the mechanical properties achieved by the static uniaxial tension tests at the elevated temperature. All the sample groups with a material thickness of 1.0 mm were included (1B, 2B, 3B, 4B).

Figure 16 shows the fracture areas and the fracture surfaces of the individual sample groups. The top row of Figure 16 shows the top view of the fractured samples where the samples of 1B, 2B, 3B were drilled in the middle of their length. As can be seen, and as expected, the samples were broken in the area of the drilled hole. The samples from group 4B were not drilled and were broken approximately in the same area as the drilled experimental samples. The bottom row of Figure 16 shows the cross section of the fractured areas. In addition, the cross section through the drilled holes can be seen on samples from the groups 1B, 2B, and 3B. The cross section of the sample 4B is for the samples that were not drilled at all. Moreover, the typical staining of the samples due to their testing at elevated temperatures can be seen here when compared to the samples evaluated at room temperature.

Table 11 gathers all the values of the mechanical properties achieved by the static uniaxial tension tests at the elevated temperature. All the sample groups with a material thickness of 1.6 mm are included (1C, 2C, 3C, 4C).

Figure 17 shows the fracture areas and the fracture surfaces of the individual sample groups. The top row of Figure 17 shows the top view of the fractured samples where the samples of 1C, 2C, 3C were drilled in the middle of their length. As can be seen, and as expected, the samples were broken in the area of the drilled hole. The samples from group 4C were not drilled and were broken approximately in the same area as the drilled experimental samples. The bottom row of Figure 17 shows the cross section of the fractured areas. In addition, the cross section through the drilled holes can be seen on the samples from the groups 1C, 2C, and 3C. The cross section of the sample 4C is for the samples that were not drilled at all. Moreover, the typical staining of the samples due to their testing at elevated temperatures can be seen here when compared to the samples evaluated at room temperature.

**Figure 18:**
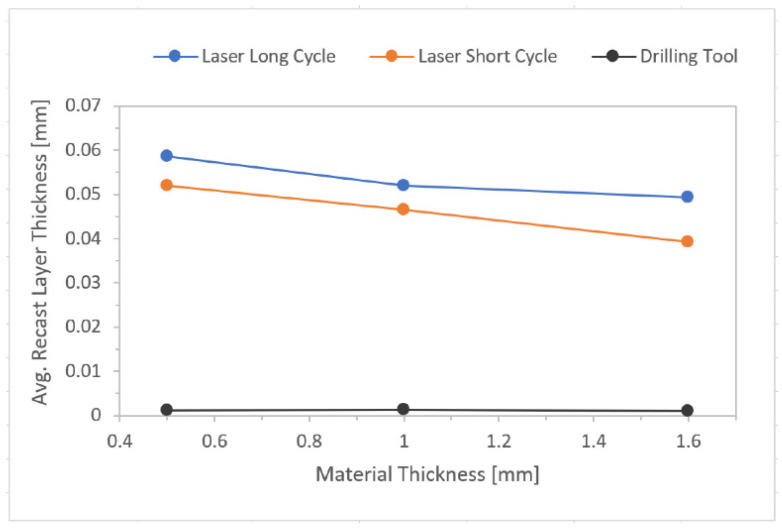
Effect of drilling methods on recast layer occurrence.

**Figure 19:**
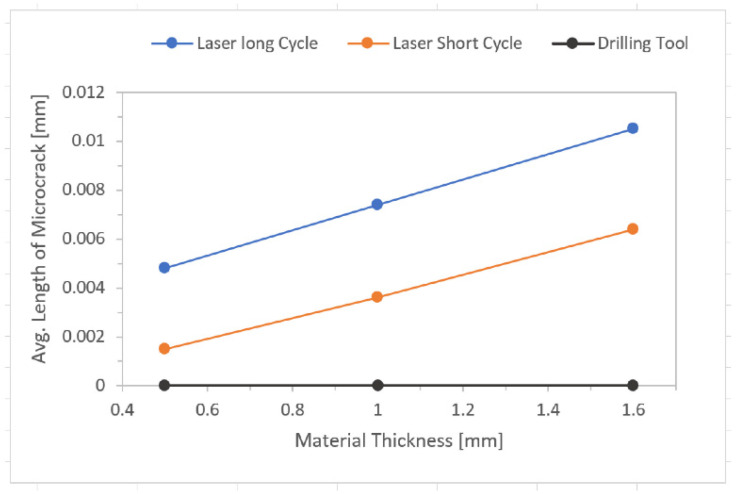
Effect of drilling methods on microcracks occurrence.

**Figure 20:**
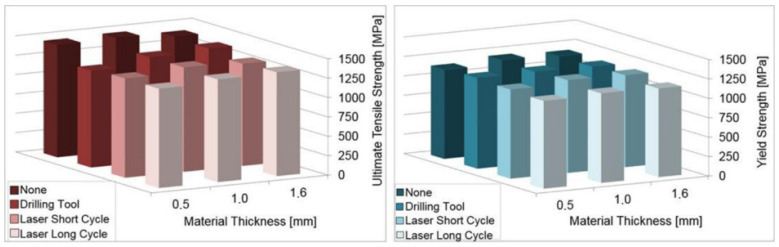
Comparison of results of uniaxial tensile testing at room temperature.

**Figure 21:**
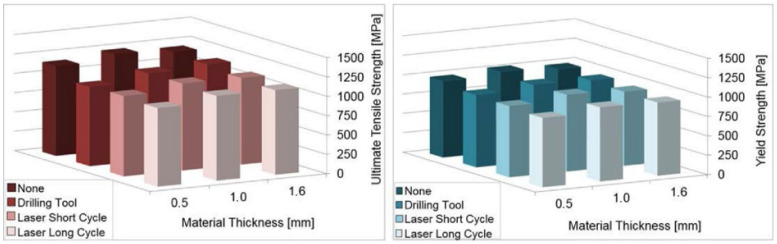
Comparison of results of uniaxial tensile testing at elevated temperature.

All the graphs (Figure 18, Figure 19, Figure 20 and Figure 21) were created based on the achieved results using Microsoft Excel for better clarity and easier comparison. For an explanation of graphs legend see Table 2. None = no hole drilled in the experimental sample. Drilling Tool = one hole drilled in the middle of the experimental sample by drilling tool and no recast layer created. Laser Short Cycle = laser drilled hole with short laser pulse width and small recast layer created. Laser Long Cycle = laser drilled hole with long laser pulse width and larger recast layer created.

### 3.3. Discussion of Achieved Results

#### 3.3.1. Formation and Average Thickness of Recast Layer (Table 4)

It was expected that on the samples drilled by the laser beam, the recast layer would be observed—whether for short or long cycle drilling. Surprisingly, a small amount of the recast layer was also observed on the samples drilled conventionally, that is, by a drilling tool. This, however, was negligible compared to the average thickness of the recast layer caused by the laser beam.

For the samples drilled by a laser long cycle it was observed that as the material thickness increased, the average thickness of the recast layer decreased. The most important thing was the fact that the highest values were always observed for laser long cycle results compared to the laser short cycle values.

For all three material thicknesses used in the experiment, it was observed that with the increasing thermal load of the base material during its drilling (highest when a laser long cycle was used), the average thickness of the recast layer increased.

#### 3.3.2. Length of Microcracks in Base Material (Table 5)

It was expected that from all three of the drilling methods used in the experiment, the highest occurrence of microcracks in the base material would be observed on the samples drilled by a laser long cycle—that is, the samples with highest values of average recast layer thickness. This was confirmed.

Microcracks were observed only on the samples that were drilled by the laser beam. There were no microcracks observed on the samples that were drilled conventionally.

It was observed that as the material thickness increased, the length of microcracks increased as well.

As the thermal load of the base material increased during its drilling (highest when a laser long cycle was used), the length of microcracks increased.

It is remarkable that there was no direct proportion between the average thickness of the recast layer and the length of the microcracks (most probably caused by the ratio of the material thickness and the drilled hole diameter). More important was the fact that the highest values were always observed in the laser long cycle results compared to the laser short cycle ones.

#### 3.3.3. Mechanical Properties of the Material Given by Uniaxial Tensile Testing (Table 6, Table 7, Table 8, Table 9, Table 10, Table 11)

It was expected that from all three of the drilling methods used in the experiment, the most significant effect on the decrease in the mechanical properties would be observed on the samples drilled by laser long cycle.

The results demonstrated that for all the material thicknesses tested, the most significant decrease in the mechanical properties was observed when the material was drilled by the laser with the long cycle than with the short cycle and/or by the drilling tool. This confirmed the initial assumptions.

As the material thickness increased, the difference in the mechanical property values was more significant.

In some cases, the values of the mechanical properties were at least comparable to the samples drilled by the drilling tools and by the laser short cycle. Based on that, some applications could benefit more from drilling by a laser (short cycle) than by a drilling tool.

When the results were compared for tensile testing at room temperature and at elevated temperatures, a 15–20% decrease in the values was observed due to the elevated temperature of the testing (Figure 20 and Figure 21).

## 4. Conclusions

- This study demonstrated that the affected areas of the base material caused by laser beam drilling, the recast layer, and microcracks in the base material have a noteworthy effect on its mechanical properties.
- A metallographic analysis of all the experimental samples provided the specific values of the average thickness of the recast layer and the length of the microcracks into the base material.
- Static uniaxial tension tests performed at room and elevated temperatures provided an overview of how much the base material was affected after its drilling mainly by laser beam.
- In real applications, where the part can be affected by thousands of drilled holes (e.g., effusion cooling holes on a combustion chamber), this could have a significant effect on the reduction in fatigue strength.

## Figures and Tables

**Table 2:** All the sample groups and the total number of experimental samples.

Drilling Method	Sample Group	Material Thickness (mm)	Number of Evaluated Samples	
			Room Temperature	Elevated Temperature
Laser Beam (long cycle = more recast layer)	1A	0.5	(23 °C)	(550 °C)
	1B	1.0	8	8
	1C	1.6	8	8
Laser Beam (short cycle = less recast layer)	2A	0.5	8	8
	2B	1.0	8	8
	2C	1.6	8	8
Drilling Tool (none recast layer)	3A	0.5	8	8
	3B	1.0	8	8
	3C	1.6	8	8
None	4A	0.5	8	8
	4B	1.0	8	8
	4C	1.6	8	8
			Sample in total: 192	

**Table 3:** Process parameters of laser beam drilling used on all applicable material thicknesses for both long and short cycles.

Variable	Unit	Sample Group					
		1A	2A	1B	2B	1C	2C
Assist Gas	-	Oxygen	Nitrogen	Oxygen	Nitrogen	Oxygen	Nitrogen
Frequency	Hz	17	18.5	16	16	13	14
Pulse Width	ms	1.5	0.5	1.2	0.5	1.5	0.5
Energy	J	10	10	11.5	10	14	13.5
Power	W	187	187	183	163	180	198
Number of Pulses	-	10	10	20	10	25	20

**Table 4:** Values of the average recast layer thickness.

Sample Group	1A	2A	3A	1B	2B	3B	1C	2C	3C
Avg. Thickness (mm) ^1^	0.0587	0.0382	0.0011	0.0520	0.0465	0.0014	0.0493	0.0393	0.0010

^1^ Each value is an average counted from the transverse and longitudinal sections, and six sections equally distributed along the hole in each section.

**Table 5:** Average values of the length of cracks in the base material.

Sample Group	1A	2A	3A	1B	2B	3B	1C	2C	3C
Avg. Thickness (mm) ^1^	0.0048	0.0015	0	0.0074	0.0036	0	0.0105	0.0064	0

^1^ Each crack observed along the hole in both, transverse and longitudinal sections, measured and average value counted for each sample group.

**Table 6:** Average values of mechanical properties—Inconel 718; material thickness 0.5 mm; tests at room temperature.

Drilling Method	SampleGroup	Samples Evaluated	E Modulus (GPa)	Max. Load (Kn)	Rp0.2 (MPa)	Rm(MPa)	A50(%)
Laser Long Cycle	1A	8	-	4.10	1127.69	1279.79	3.51
Laser Short Cycle	2A	8	-	4.10	1146.80	1279.92	3.48
Drilling Tool	3A	8	-	3.93	1166.97	1248.44	2.41
None	4A	8	-	4.58	1146.10	1453.30	14.08

Note: E Modulus was not evaluated by the test cell software.

**Table 7:** Average values of mechanical properties—Inconel 718; material thickness 1.0 mm; tests at room temperature.

Drilling Method	Sample Group	Samples Evaluated	E Modulus (GPa)	Max. Load (kN)	Rp0.2 (MPa)	Rm (MPa)	A50 (%)
Laser Long Cycle	1B	8	-	8.07	1151.57	1323.34	4.42
Laser Short Cycle	2B	8	-	8.35	1192.80	1347.42	5.18
Drilling Tool	3B	8	-	8.22	1173.82	1346.97	4.96
None	4B	8	-	8.95	1197.12	1467.40	15.03

Note: E Modulus was not evaluated by the test cell software.

**Table 8:** Average values of mechanical properties—Inconel 718; material thickness 1.6 mm; tests at room temperature.

Drilling Method	Sample Group	Samples Evaluated	E Modulus (GPa)	Max. Load (kN)	Rp0.2 (MPa)	Rm (MPa)	A50 (%)
Laser Long Cycle	1C	8	-	13.07	1140.12	1339.09	5.14
Laser Short Cycle	2C	8	-	13.07	1182.23	1317.55	5.91
Drilling Tool	3C	8	-	13.51	1165.50	1384.40	7.71
None	4C	8	-	13.97	1173.86	1407.76	10.53

Note: E Modulus was not evaluated by the test cell software.

**Table 9:** Average values of mechanical properties—Inconel 718; material thickness 0.5 mm; tests at elevated temperature.

Drilling Method	Sample Group	Samples Evaluated	E Modulus (GPa)	Max. Load (Kn)	Rp0.2 (MPa)	Rm (MPa)	A50 (%)
Laser Long Cycle	1A	8	147.24	3.30	893.39	1013.99	2.03
Laser Short Cycle	2A	8	140.57	3.39	917.29	1037.96	2.57
Drilling Tool	3A	8	144.84	3.31	931.96	1023.41	1.90
None	4A	8	147.24	3.30	893.39	1013.99	2.03

**Table 10:** Average values of mechanical properties—Inconel 718; material thickness 1.0 mm; tests at elevated temperature.

Drilling Method	Sample Group	Samples Evaluated	E Modulus (GPa)	Max. Load (kN)	Rp0.2 (MPa)	Rm (MPa)	A50 (%)
Laser Long Cycle	1B	8	146.22	6.80	954.50	1095.89	2.54
Laser Short Cycle	2B	8	148.55	6.99	988.60	1122.57	2.64
Drilling Tool	3B	8	145.40	6.91	987.98	1123.01	3.45
None	4B	8	170.53	7.68	1030.69	1237.19	8.42

**Table 11:** Average values of mechanical properties—Inconel 718; material thickness 1.6 mm; tests at elevated temperature.

Drilling Method	Sample Group	Samples Evaluated	E Modulus (GPa)	Max. Load (kN)	Rp0.2 (MPa)	Rm (MPa)	A50 (%)
Laser Long Cycle	1C	8	142.05	11.05	942.76	1092.65	2.57
Laser Short Cycle	2C	8	143.34	11.01	953.68	1117.86	3.29
Drilling Tool	3C	8	150.29	11.46	971.40	1163.57	6.64
None	4C	8	165.71	11.85	988.67	1193.20	7.34

## Data Availability

Not applicable.
